# Outreach for chlamydia and gonorrhoea screening: a systematic review of strategies and outcomes

**DOI:** 10.1186/1471-2458-13-1040

**Published:** 2013-11-04

**Authors:** Belinda Hengel, Muhammad S Jamil, Jacqueline K Mein, Lisa Maher, John M Kaldor, Rebecca J Guy

**Affiliations:** 1 Apunipima Cape York Health Council, Cairns, Australia; 2 Kirby Institute, University of New South Wales, Sydney, Australia

**Keywords:** Sexually transmissible infections, Outreach, Testing, Systematic review, Chlamydia

## Abstract

**Background:**

High *Chlamydia trachomatis* (CT) and *Neisseria gonorrhoeae* (NG) prevalence have been reported in populations that do not regularly access health centres for sexually transmissible infections (STI) testing. We reviewed current outreach strategies used to increase access to STI testing and their outcomes.

**Methods:**

We systematically reviewed the literature for English language studies published between 1 January 2005 and 28 January 2011 describing CT and/or NG screening programs in non-clinical outreach settings.

**Results:**

We identified 25 programs, with the majority occurring in either Australia (32%) or the United States (32%). The most common target groups were young people aged 15–29 years (52%), men who have sex with men (24%) and sex workers (8%). The median CT positivity was 7.7% (Inter Quartile Range [IQR]: 3.0%-11.1%, n=19 programs), and median NG positivity was 2.6% (IQR: 0.0%-8.0%, n=10). The median participation rate was 53% (IQR: 23.9%-81.3%), and a median of 79.6% (IQR: 55.1%-89.4%) of participants were tested, with a median of 100 tests conducted per program (IQR: 65–331, range: 11–1808). Across all settings the participation rate was highest among target groups gathering in community service venues (community centres, parenting centres, homeless shelters) (median=81.4%, n=4), and social venues (sporting venues or bars) (80.4%, n=1). Lower participation rates were found in street/public community areas (median=23.9%, n=3) and sex on premises venues (10.4% and 24.3%, n=2).

**Conclusions:**

The review indicated that although CT and NG outreach programs reached a relatively small number of people the yield of infections is high. Settings which appear to be more effective at encouraging participation appear to be those within an existing venue, rather than in public areas.

## Background

*Chlamydia trachomatis* (CT) is the most commonly reported notifiable infectious disease in the United States, Australia and many European countries with notifications increasing steadily each year [1-3]. High CT and *Neisseria gonorrhoeae* (NG) prevalence’s of 3-10% have been reported in young people, men who have sex with men (MSM) and sex workers [4-6].

Screening and treatment for sexually transmissible infections (STIs) is an important prevention strategy as untreated genital CT and NG infection can lead to pelvic inflammatory disease [7,8] and infertility [9]. Traditionally, screening has relied heavily on individuals self-presenting to clinical services. However, many populations fail to regularly access health centres for STI testing [10,11] due to barriers such as lack of services and transport [12,13], stigma [13], confidentiality concerns [12,14-16], cost [12,17], and lack of knowledge and awareness about STIs [14,18]. Offering screening for CT and NG outside clinical settings is often conducted to reach populations at risk of STIs with poorer health seeking behaviour. The availability of nucleic acid amplification technology (NAAT) since the mid-1990s and the ability to test urine and self-collected swabs for CT and NG has also made it more feasible to conduct screening in a non-invasive manner in non-clinical settings [19].

Over the past few years a body of observational research has accrued on outreach based STI screening programs. We review studies published since 2005 to describe and contrast current strategies used in outreach programs, the testing uptake achieved and outcomes of testing. To our knowledge, this is the first systematic review to synthesise findings of outreach STI screening programs based on testing coverage, yield and costs.

## Methods

This review was conducted in accordance with guidelines outlined in the PRISMA statement [20].

### Review strategy

Electronic bibliographic databases PubMed and EMBASE were searched for English language published studies between 1 January 2005 and 28 January 2011. We used the following key search terms: Chlamydia, or Chlamydia Infections, or Chlamydia trachomatis, or Gonorrhoea, AND Screening or Mass Screening or Testing. Reference lists, where relevant were screened for related studies. The search terms were broad as this review formed part of a larger review focused on testing in a range of non-clinical settings.

We defined outreach as an activity undertaken in order to offer CT / NG screening to target groups who may have difficulty accessing existing services. The papers and information extracted were independently reviewed by two authors [MJ and BH]. Disagreements were resolved through discussion and consensus obtained. A paper was included if it described an outreach program which was: led by an external organisation and offered CT and/or NG screening in person to populations in the community, street or at a specific venue (social venues, sex venues or community organisations); the screening activity was primarily focused on increasing access to STI testing among populations at risk for STIs; and the study reported at least the number of STI tests conducted. Studies were excluded for the following reasons: the outreach program was undertaken in an institution where daily attendance was compulsory such as a school, workplace or detention centre; the testing occurred in a clinical setting where STI testing routinely took place; postal screening kits were mainly used; screening was conducted within a cohort study or a randomised controlled trial; the study was designed to estimate STI prevalence; anonymous testing was conducted with results and treatment withheld from participants; specimens were not self-collected; and no original data were presented e.g. review, editorial.

For the studies which met the inclusion criteria, information was extracted on the target group, setting, infections tested for, recruitment details, incentive use, specimen collection method, type of specimens collected, test results and case and partner notification method. Quantitative data were also extracted on the number of people invited who agreed to participate, the number of participants tested, the number of positive CT and NG tests, the number of individuals notified of their results and treated after a positive test, the number sexual partners which were notified of possible contact with a STI and treated, and any information on costs.

The following definitions were used throughout the review:

i. Participants: people who were approached and agreed to participate in the screening activity

ii. Participation rate: the number of participants divided by the number of people approached

iii. Testing rate: the number of specimens collected divided by the number of participants

iv. Positivity: the percentage of tests which were positive

v. Results notification rate: the percentage of people with a positive test who were informed of their results

vi. Treatment rate: the proportion of people with positive result who were treated.

### Analysis

We conducted a frequency analysis of the outcomes extracted. For the participation rate, testing rate and positivity outcomes we calculated the median and inter-quartile range (IQR) across programs. We described the overall outcomes for all programs included in the review, then focused primarily on the participation and testing rates for each different setting type and population group, including; young people (age 15–29 years), MSM, sex workers based outside of registered brothels, travellers in hostels, men living in temporary settlements in South Africa, clients of homeless shelters, and attendees of community centres. Setting types were grouped as; (i) community or street based and (ii) venue based outreach. Venue based outreach was then further divided into; (i) sex venues, (ii) community services venues and (iii) social venues (including bars and sporting venues).

## Results

### Overview

**Figure 1:**
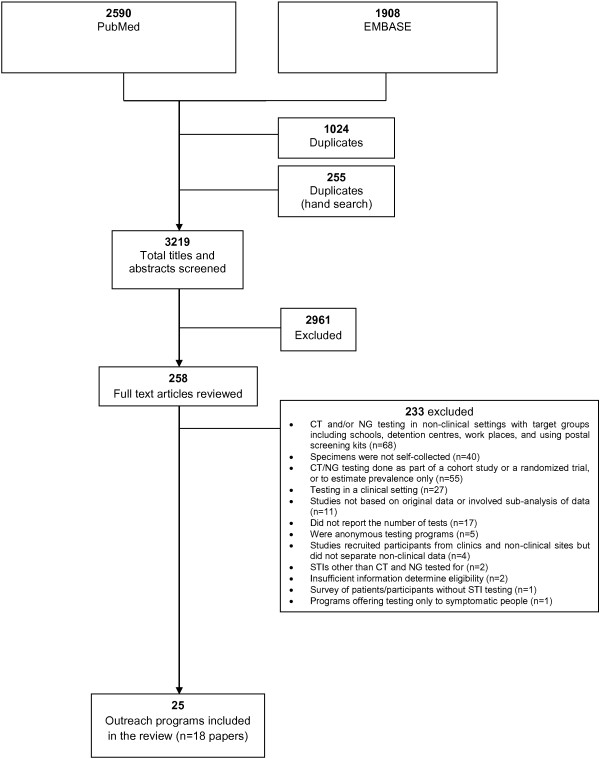
Search strategy.

The initial search identified 3219 unique articles for which the titles and abstracts were reviewed; 3201 were excluded leaving 18 papers in the review (Figure 1).

The 18 papers described 25 outreach programs undertaken in a range of countries and settings. Nearly half of program targeted youth age 15–29 years (52%) followed by MSM (24%) then female sex workers (8%). Other groups included: attendees of community centres, travellers staying in hostels, clients of homeless shelters and settlement dwellers in South Africa (Table 1). Participants were recruited from a variety of settings: street or community areas (28%), community services (28%), social venues (24%) and sex venues (20%).

**Table 1:** Summary of CT and NG outreach programs (n=25)

**Category**	**Sub-category**	**Studies**	
		**n**	**%**
Country/region	America/Canada	10	40%
	Asia/Africa	3	12%
	Australia/New Zealand	8	32%
	Europe/Scandinavia	2	8%
	United Kingdom	2	8%
Target gender	Female	2	8%
	Female/transgender	1	4%
	Male	10	40%
	Male/female	12	48%
Target setting	Social venue	6	24%
	Community service venue	7	28%
	Sex Venue	5	20%
	Street or community area	7	28%
Target group	Youth	13	52%
	Men who have sex with men	6	24%
	Community centre clients	1	4%
	Shelter clients	1	4%
	Travellers in budget hostels	1	4%
	Settlement dwellers	1	4%
	Sex Workers	2	8%
Incentive provided	Monetary	1	4%
	Non-monetary	8	32%
	No	6	24%
	Not stated	10	40%
Infections tested for	CT only	10	40%
	CT/others	1	4%
	CT/NG	8	32%
	CT/NG/others	6	24%
Specimen type collected	Urine	17	68%
	Urine/recto-anal swab	3	12%
	Urine/vaginal swab	5	20%

The majority of programs offered screening for CT only (40%), CT / NG only (32%), and HIV and/or syphilis in addition to CT / NG (28%). Of the 25 programs the median number of tests conducted was 100 (IQR: 65 – 331, range: 11–1808). Participation rates were documented in ten programs, with a median of 53% (IQR: 23.9% - 81.3%, range: 10.4% - 96.7%). Testing rates were documented in 14 programs, with a median of 79.6% (IQR: 55.1% - 89.4%, range: 21.3% - 98.6%). Nineteen programs reported CT positivity with a median of 7.7% (IQR: 3.0% - 11.1%). The median NG positivity in ten programs was 2.6% (IQR: 0.0% - 8.0%).

Of the 25 programs, nine (36%) described using incentives to encourage participation (eight were non-monetary including movie vouchers, condoms, food coupons and one involved cash), ten programs did not specify incentive use and a further six programs did not use incentives. Seven programs documented the result notification rate, with a median of 100% (IQR: 81% - 100%). These programs used a combination of in-person, phone, SMS, mail and email to provide participants with their results. Treatment rates were documented in eight programs, the majority (75%) of these documented treatment rates of 100%. No programs documented partner notification outcomes.

Program costs were available in four programs. Morris et al. calculated costs per CT test taken and cases detected in two Californian youth programs in street settings and parenting centres. Costs were calculated by dividing hours worked by number of tests taken and the number of positive CT tests. Street settings took 2.3 person hours per test and 43 hours per case detected. Parenting centres took 0.9 person hours per test and 13.5 hours per case detected. To increase participation peer volunteers were used in a number of screening sites, however volunteer hours worked were not included in the overall cost calculations [21]. Buhrer-Skinner et al. documented crude costs per test (AUD$26.20) and case detected (AUD$431.80) for outreach targeting youth, defence personnel and travellers staying in hostels. This is compared to in-house laboratory costs of AUD$25.90 per test and AUD$291.60 per case detected. Staff time, transport and set up costs were not included in estimates [22].

### Young people

Of the 13 youth outreach programs the majority were conducted in Australia (38%) and the United States (US) (38%) (Table 2). Common settings were street/community areas (38.5%), community service venues (38.5%) or social venues (23%). The median number of tests per program was 74 (IQR: 49 – 331). The median participation rate was 79.6% (IQR: 23.9% - 81.3%, range: 21.3% - 81.7%) (n=7) and the median testing rate was 79.6% (IQR: 48.8% - 79.6%, range: 21.3% - 85.2%) (n=7). The median CT positivity was 6.3% (IQR: 3.9 - 9.5%) (n=9), NG positivity was documented by two programs (0.0% and 1.2%).

**Table 2:** Outcomes of CT and NG outreach programs targeting youth (n=13)

**Author, year**	**Country**	**Target group**	**Target setting**	**Participation rate n (%)**	**Specimen return method**	**Specimens returned (n)**	**% tested (%)**	**CT positive**		**NG positive**		**Treatment rate n (%)**
								**n**	**% (95% CI)**	**n**	**% (95% CI)**	
Buhrer-Skinner, 2009, [22]	Australia	School leavers	Street or community area	68 (21.3%)	In person	68	21.3%	0	0 (0–5.3)	.	.	.
		Youth at risk of dropping out of school	Service venue	23	In person	23	.	3	13% (2.8-33.6)	.	.	3 (100%)
Gold, 2007, [23]	Australia	Players at a football club	Social venue	108	In person	92	85.2%	3	3.9% (0.8 – 11)	0	0 (0–4)	.
Gotz, 2006 [24]	Netherlands	Young people, particularly of non-Dutch ethnicity	Street or community area	79 (27.5%)	In person, or post return	49	62%	6	12.2% (4.6 – 24.8)	.	.	6 (100%)
		Groups of new immigrants and, teenage school dropouts	Service venue	76 (81.7%)	In person, or post return	74	79.6%	7	9.5% (3.9 – 18.5)	.	.	7 (100%)
Johnson, 2008 [25]	USA	Young people appearing in the Family Courts	Service venue	1808 (79.6%)	In person	1808	79.6%	124	7.8% (6.5 -9.2)	19	1.2% (0.7 – 1.9)	128 (96%)
Kong, 2009 [26]	Australia	Young people in sporting clubs	Social venue	709	In person	709	.	28	3.9% (2.6 -5.7)	.	.	28 (100%)
Lorimer, 2009 [27]	UK	Young people attending a leisure centre	Social venue	127 (80.4%)	In person	62	48.8%	.	.	.	.	.
Martin, 2009 [28]	Australia	Young people at multiple venues	Street or community area	204	In person or drop off return	150	79.6%	.	.	.	.	.
Morris, 2010 [21]	USA	Young people in multiple settings	Street or community area	.	In person or post	.	.	31	4.9% (3.4 – 6.9)	.	.	.
		Parenting centres	Service venue	.	In person or post	.	.	21	6.3% (4 – 9.5)	.	.	.
Marrazzo, 2007 [29]	USA	Men at street venues	Street or community areas	11 (23.9%)	In person	.	.	.	.	.	.	.
		Men attending a drug treatment facility	Service venue	26 (81.3%)	In person	.	.	.	.	.	.	.

The highest participation rate (81.7%) (tests=74) was reported by Gotz et al. involving outreach among new immigrants, school dropouts and people from vocational schools who met regularly at a community service venue in the Netherlands [24]. A high participation (81.3%) rate was also reported by Marrazzo et al. (tests=26) among young men attending drug treatment centres [29].

Johnson et al. also reported a high participation rate (79.6%) (tests=1808) in a program in the US family court system where collection of a urine sample was mandatory for drug testing. Young people were asked to consent to a small portion of this urine sample being tested for STIs [25].

The lowest youth participation rate was in a program targeting youth at a festival (21.3%) (tests=68) where health staff held a stall offering STI testing [22]. Marrazzo et al. and Gotz et al. also reported low participation rates of 23.9% (tests=11) and 27.5% (tests=49) respectively, during outreach in community/street settings.

The highest testing rate (85.2%) (tests=92) was found by Gold et al. in a program in Australia where football clubs agreed to have screening offered in club rooms after training [23]. High testing rates were also reported by Gotz et al. (79.6%) in the program targeting new immigrants, school dropouts and people from vocational schools, and Johnson et al. (79.6%) in the court outreach program. Martin et al. also reported a high testing rate of 79.6% (tests=150) where young people were approached at dance parties, the beach and music events in Australia, and had the option of posting a kit back [28].

### MSM

The six programs targeting MSM were conducted in Australia, the US, Canada and the United Kingdom (UK), mainly within sex on premises venues (83%) [30-33] (Table 3). The median number of tests per program was 175 (IQR: 144–521), the median testing rate was 72.3% (IQR: 48.1% - 92.9%, range: 41.1% - 96.5%) (n=4), the median CT positivity was 2% (IQR: 0.6% - 7.1%) (n=4), and the median NG positivity was 0.5%, (IQR: 0% - 11.1%) (n=3).

**Table 3:** Outcomes of CT and NG outreach programs targeting MSM, sex workers and other populations (n=12)

**Author, year**	**Country**	**Target group**	**Target setting**	**Participation rate n (%)**	**Specimen return method**	**Specimens returned (n)**	**% tested (%)**	**CT positive**		**NG positive**		**Treatment rate n (%)**
								**n**	**% (95% CI)**	**n**	**% (95% CI)**	
Blank, 2005 [34]	USA	MSM in bars and clubs	Social venue	.	In person	183	41.1%	2	1.1% (0.1 -3.9)	1	0.5% (0 – 3.0)	.
Buhrer-Skinner, 2009 [22]	Australia	Travellers staying in backpacker accommodation	Social venue	65	In person	65	.	5	7.7% (2.5 – 17.0)	.	.	5 (100%)
Emerson, 2010 [30]	UK	MSM attending two venues	Sex venue	173	In person	.	96.5%	5	3% (1.0 – 6.8)	.	.	.
Grimley, 2006 [35]	USA	People at homeless shelters	Service venue	416 (96.7%)	In person	.	98.6%	32	10.8% (7.5-14.9)	12	4.1% (2.1-7.0)	40 (91%)
Lewis, 2008 [36]	South Africa	Settlement dwellers	Street or community area	309	In person	301	97.4%	26	8.6% (5.7 – 12.4)	19	6.3 (3.8-9.7)	.
Lister, 2005 [31]	Australia	MSM attending four sex on premises venues	Sex venues	161 (10.4%)	In person	.	89.4%	16	11.1% (6.5 -17.4)	16	11.1% (6.5 – 17.4)	. (100%)
		MSM attending four sex on premises venues	Sex venues	521 (24.3%)	In person	.	.	.	.	.	.	.
McNeely, 2010 [32]	USA	MSM attending bars and clubs were sex takes place	Sex venue	1694	In person	934	55.1%	.	.	.	.	.
O’Byrne, 2008 [33]	Canada	MSM attending two bathhouses	Sex venue	.	Pick up/drop off	52	.	0	0 (0 – 7.4)	0	0 (0–7.4)	.
Rusch, 2008 [37]	Canada	Women and transgender at a community centre	Service venue	126	In person	92	73%	2	2.2% (0.3 – 7.9)	0	0 (0–4.0)	.
Wi, 2006 [38]	Philippines	Street based female sex workers (FSW)	Street or community area	100	In person	100	.	35	35% (25.7–45.2)	23	23% (15.2– 32.5)	.
		FSW in Karaoke bars	Social venue	100		100	.	18	18% (11 – 26.9)	8	8% (3.5 – 15.2)	.

Participation rates were documented in two programs, both by Lister et al. at 10.4% (tests=144) and 24.3% (tests=521). The authors compared two systems of outreach in the same venues over consecutive years to describe if a comprehensive program compared to an anonymous program increased clients receiving results and treatment. The anonymous program did not collect any identifying information from participants, instead participants were given a card with a unique study identifier and a phone number to call for their results [39]. The comprehensive program was appointment based and client contact details were collected. Lister et al. found the comprehensive program accessed fewer men per hour but the CT/NG positivity was higher among those who obtained their results, compared with the anonymous program [31].

Testing rates were documented in five programs. Emerson et al. reported the highest testing rate (96.5%) (tests=167) in a program which targeted MSM at two sex on premises venues in the UK and involved offering testing monthly [30]. Lister et al. as described above, also reported a high testing rate of 89.4% (tests=144) in their comprehensive screening program. The lowest testing rate (41.1%) (tests=183) was reported by Blank et al. in a program offering STI screening along with a full health check at MSM attending bars in New York. This program used entertainment staff and volunteers to promote screening [34].

### Sex workers

Two outreach programs, both reported by Wi et al. targeted sex workers. The programs were designed to increase access to preventative health care for street based sex workers and guest relations officers in karaoke bars in the Philippines [38]. Testing and participation rates were not documented. The CT and NG test positivity varied greatly between settings with those screened in karaoke bars having a CT and NG positivity of 18% and 8%, compared to 35% and 18% among street based workers, respectively. The numbers of sex workers screened in both programs was the same (100 participants each). Peer educators were used to reach a broader group of sex workers in both settings. Outreach staff also requested that participants complete a questionnaire covering demographics and behavioural questions.

### Settlement dwellers

Lewis et al. described an outreach program targeted at men living in temporary settlements in South Africa. The program was requested by the local male residents and involved a mobile van offering STI and HIV screening fortnightly, with participants completing a brief demographic and behavioural questionnaire prior to testing. A total of 301 men were tested, the participation rate was not reported, however the testing rate was 97.4%. The CT positivity was 8.6% and NG positivity was 6.3% [36].

### Homeless shelters

The highest participation (96.7%) and testing rates (98.6%) (tests=410) in the review were found in an outreach program targeting clients of homeless shelters. The CT positivity was 10.8% and NG positivity was 4.1%. Nearly all participants (98%) were informed of their results and 91% of those testing positive were treated. Study staff provided treatment at the homeless shelter, and participants unable to be located for treatment were referred to the health department for follow-up [35]. Participants received a food voucher as an incentive to participate.

### Travellers in hostels

Buhrer-Skinner et al. offered screening to travellers staying in budget hostels. Outreach staff provided an education session in the evening then offered STI testing at the completion of the session. Of the 65 tests conducted the CT positivity was 7.7%. Participation and testing rates were not documented.

### Community centre attendees

Rusch et al. offered STI screening to a group of women and transgender people (approximately half were sex workers) attending a community venue. STI screening occurred during a weekly program which provided food and general health care. Participants were offered $10 for participating and completing a questionnaire describing demographics, drug use, sexual activity and health care access. Participation rates were not recorded, however the program reported a testing rate of 73% (tests=92), the CT positivity was 2.2% and no NG cases were detected [37].

## Discussion

This review indicates that since 2005 outreach programs for CT and NG have been conducted in a range of populations across a wide variety of settings. Although the programs reached a relatively small number of people, the yield of infections was high. Settings which appear to be more successful at encouraging participation were those where screening was offered within an existing venue (community centre, homeless shelter or parenting centre) or sporting club, rather than on the street or public community areas.

The strength of this review is that we included programs from a variety of countries and population subgroups to increase the generalisability of the findings. This review also has some limitations. First, we did not search the grey literature so it is possible that other unpublished outreach programs were not identified. Second, we restricted the search to 2005 to focus on the current outreach methods using NAAT. Third, due to the heterogeneity of the programs and outcomes we were unable to conduct a meta-analysis. Fourth, many of the programs did not provide the necessary data to calculate participation or testing rates. Fifth, search criteria did not include any cost studies reported which related to the programs included in this review. Finally, it is difficult to disentangle the elements of the programs which may have resulted in high participation rates.

The highest participation rates appeared to be in venues providing a community service, and social venues such as sporting clubs and bars, whereas the lowest participation rates were in the street or public community areas and male sex venues. The relatively higher participating rate in community service venues or sporting clubs may be due to a number of factors. Recruitment in venues would be supported by the venue managers, and thus potential participants may feel more comfortable participating where trusted and known venue staff may be more active in assisting with recruitment. Second, as the venue is a closed environment participants would see others participate which would reassure them about their own involvement.

It is possible that the approach, type of staff, use of peers and incentives used in programs may also influence the participation rate however there were limited information in many studies to explore this formally. A third of programs included in this review specified the use of peers in their outreach, with Morris et al. showing the use of peer volunteers increased chlamydia case detection by 3.2% (CI 1.3 – 7.8) compared to outreach where peer volunteers were not used [21]. Incentives also appear to be commonly used in outreach programs; as we excluded randomised control trials (RCT) their effectiveness was not evaluated in any of the studies in this review. However a number of RCTs have found monetary incentives lead to an increase in the uptake of preventative health care [40]. In an education setting, Currie et al. reported higher participation rates in CT screening programs using monetary incentives and SMS to promote the event compared to non-monetary incentives [41]. Further research is needed to evaluate the effectiveness of incentives to increase participation in outreach screening programs across various settings.

Outreach programs require significant effort and labour to implement [21]. Our review demonstrated that outreach screened a relatively small number of people, but the yield of CT and/or NG infections was high. Understanding efficiency and costs is an important part of program evaluation, however very few programs documented efficiency of their outreach program in terms of costs. Of the few that did, Morris et al. calculated costs in terms of staffing hours and reported less costs per test taken and case detected when outreach was undertaken within an existing venue, compared to street based outreach.

In addition to the testing uptake by the target group, the success of screening depends on treatment rate. Seven programs documented the rate of notification of results with a median of 100% being notified. Outreach programs included in this review used a combination of methods (phone, email and SMS) to give participants their results, innovative methods are often required as the initial physical contact may be the only one the service has with the participant. This contrasts with clinical and educational settings, where there is greater opportunity to provide results as the client may be a regular patient of the clinic or in the case of students they attend schools daily, and there is often a school clinic which they can attend at times convenient to them, and thus can be followed up relatively easily [42].

To maximise the benefits of outreach, programs should specifically target groups at risk of STIs who have limited access to sexual health services. The key aim of an outreach initiative should be to respond to an unmet need, rather than simply trying to maximise testing numbers. Most programs in the review targeted such populations, however only 60% of the programs actually recorded the health seeking behaviour of the target groups.

## Conclusions

Outreach STI testing programs were established in a range of settings and many targeted populations with a high yield of infections. Therefore in populations and areas where access to sexual health services is limited, supplementing existing clinical services with strategies such as outreach programs is worth considering. The review provides some insight into strategies which can maximise the participation and testing rates and yield of infections in outreach programs, and also methods for providing treatment outside of clinical settings.

## Competing interests

The authors declared that they have no competing interests.

## Authors’ contributions

BH undertook the analysis of results obtained through the systematic review and drafted the manuscript, MJ undertook the systematic review, JM and LM edited the manuscript, JK edited the manuscript and provided advice with the design of the analysis, RG provided overall guidance with the analysis design and assistance in drafting the manuscript. All authors read and approved the final manuscript.

## Pre-publication history

The pre-publication history for this paper can be accessed here:

http://www.biomedcentral.com/1471-2458/13/1040/prepub
